# Genome analysis of *Actinobacillus pleuropneumoniae* strain APPFJLYC01 reveals multidrug resistance and high virulence potential

**DOI:** 10.1371/journal.pone.0336060

**Published:** 2025-11-14

**Authors:** Zhihong Fang, Zecheng Lin, Chuchu Duan, Xiaojin Liu, Zhongfeng Luo, Cuiqin Huang, Xiaohua Li, Xintian Zheng

**Affiliations:** 1 College of Life Science, Longyan University, Longyan, China; 2 College of Animal Science, Fujian Agriculture and Forestry University, Fuzhou, China; 3 Fujian Provincial Key Laboratory of Animal Infectious Disease Control and Biotechnology, Longyan, China; 4 Engineering Research Center of Zoonotic Diseases Prevention and Control, Fujian Universities, Longyan, China; Federal University Dutse, NIGERIA

## Abstract

*Actinobacillus pleuropneumoniae* is the primary etiological agent of porcine contagious pleuropneumonia, a devastating respiratory disease that causes substantial economic losses to the global swine industry. The emergence of multidrug-resistant strains with enhanced virulence poses increasing challenges to disease control and necessitates comprehensive genomic characterization to inform targeted intervention strategies. This study aimed to characterize the genomic features, antimicrobial resistance profile, and pathogenic potential of a novel A. pleuropneumoniae strain isolated from a severe outbreak in China, with particular focus on identifying unique resistance mechanisms and virulence determinants. We isolated strain APPFJLYC01 from lung and bronchial tissues of pigs during a severe pleuropneumonia outbreak in Fujian Province, China (incidence rate 30%, mortality rate 56%). Complete genome sequencing was performed using combined PacBio Sequel II and DNBSEQ platforms, followed by comprehensive bioinformatics analysis including virulence factor identification, antibiotic resistance gene profiling, phylogenetic analysis, and comparative genomics. Pathogenicity was evaluated through experimental infection of 3-week-old piglets with subsequent clinical, pathological, and histopathological examinations. The genome of APPFJLYC01 strain is 2,308,741 bp in size, encoding 2,149 genes. Notably, it contains 190 virulence factor homologs and 10 resistance genes. Phylogenetic analysis based on CorePan revealed that APPFJLYC01 shares a close evolutionary relationship with strain JL03, potentially due to their shared geographical origin in China. Pathogenicity evaluation in piglets confirms its high virulence. These findings highlight APPFJLYC01 as a multidrug-resistant and highly virulent strain, providing insights for controlling porcine pleuropneumonia.

## 1 Introduction

Actinobacillus pleuropneumoniae (*A. pleuropneumoniae*) is a Gram-negative bacterium and the primary etiological agent of porcine contagious pleuropneumonia. This severe respiratory disease has caused substantial economic losses to the global swine industry [1–3]. Characterized by an explosive epidemic pattern, it often presents as fibrinous, hemorrhagic, and necrotizing pneumonia. The incidence of porcine pleuropneumonia ranges from 8% to 100%, with mortality rates varying between 4% and 100% [4].

To date, 19 serovars of *A. pleuropneumoniae* have been identified based on capsular antigens, and two biotypes have been recognized, exhibiting significant variations in virulence and geographical distribution [5–7]. In China, serovars 1, 3, 4, 5, and 7 are the most prevalent [8]. The two biotypes are differentiated by their dependence on nicotinamide adenine dinucleotide (NAD) for growth [9].The virulence of *A. pleuropneumoniae* is attributed to multiple factors, including Apx exotoxins, capsular polysaccharides (CPS), lipopolysaccharides (LPS), outer membrane proteins, iron-acquisition systems, and adhesins [10–12].

In most regions worldwide, antibiotics remain the most effective intervention for reducing mortality and controlling clinical disease during outbreaks of *A. pleuropneumoniae*. Antibiotic therapy is typically administered at the onset of symptoms to prevent mortality and limit the spread of infection. However, the emergence of strains with varying levels of antibiotic resistance has become a growing concern in recent years [13,14]. Numerous studies have demonstrated increasing numbers of *A. pleuropneumoniae* with acquired resistance to tetracycline, in addition to increasing levels of resistance to penicillin, amoxicillin, ampicillin, and macrolides [14–16]. These results highlighted the unmet needs for disease control and a comprehensive understanding of antimicrobial resistance in *A. pleuropneumoniae*.

Vaccination is a common strategy for preventing porcine pleuropneumonia, and commercial vaccines targeting specific serovars are available [3,17]. However, the lack of cross-protective immunity among serovars, coupled with the geographical variation in dominant serotypes, poses significant challenges for vaccine development [18,19]. Despite the availability of several vaccines, the genetic and phenotypic diversity of *A. pleuropneumoniae* serovars complicates the development of a broadly protective vaccine [20,21].

In mid-April 2023, an outbreak of *A. pleuropneumoniae* infection occurred on a pig farm with over 300 sows in Fujian Province, China. The outbreak primarily affected 70-day-old pigs, resulting in an incidence rate of 30% and a mortality rate of 56%. Clinical signs in infected pigs included loss of appetite, elevated body temperature, dyspnea, and the presence of red, foamy nasal discharge with blood in acute cases. A Gram-negative bacterial strain was isolated from the lungs and bronchi of infected pigs.

This study aimed to elucidate the genetic characteristics of the isolated strain, including its antimicrobial resistance and virulence gene profiles, through whole-genome analysis. The findings are expected to provide valuable insights for the selection of appropriate vaccines and antimicrobial agents to control *A. pleuropneumoniae* infections.

## 2 Methods

### 2.1 Sample collection and animal sources

Samples for bacterial isolation were collected from the lungs and bronchi of pigs infected with *A.pleuropneumonia* on a farm in Fujian Province, China. For pathogenicity testing, ten 3-week-old piglets were purchased from a farm in Longyan City, Fujian Province. Prior to experimentation, the piglets were confirmed to be negative for African swine fever virus, classical swine fever virus, porcine reproductive and respiratory syndrome virus (PRRSV), and pseudorabies virus (PRV) antigens. All animal experiments were approved by the Animal Ethics Committee of Longyan University (Approval No. LY2024014L) and conducted in compliance with the guidelines for the care and use of laboratory animals. Piglets were housed in a controlled environment with a temperature of 22–25°C, relative humidity of 50–60%, and ad libitum access to food and water.

### 2.2 Bacterial isolation and identification

*A. pleuropneumoniae* was isolated from the lungs and bronchi of pigs. Isolation and identification of the bacterial strain were conducted at the Engineering Research Center of Zoonotic Diseases Prevention and Control, Fujian Universities, using standard microbiological techniques. *A. pleuropneumoniae* was isolated on chocolate agar supplemented with 5% sheep blood and incubated at 37°C under 5% CO₂ for 24–48 hours. The identity of the isolate was confirmed by PCR targeting the species-specific *apxIV* gene [22].

### 2.3 Serotyping

Serotype identification was performed using the agar gel diffusion method, following established protocols. The analysis was carried out by Zhaofenghua (Beijing) Biotechnology Co., Ltd.

### 2.4 Genome sequencing and assembly

The genome of *A. pleuropneumoniae* strain APPFJC001 was sequenced using a combination of PacBio Sequel II and DNBSEQ platforms at the Beijing Genomics Institute (BGI, Wuhan, China). For PacBio sequencing, four SMRT cells with Zero-Mode Waveguide arrays were used to generate subreads. Subreads shorter than 1 kb were removed, and the remaining reads were self-corrected using Canu. Draft genomic unitigs were assembled from the high-quality corrected circular consensus sequence subreads. To enhance sequence accuracy, single-base corrections were made using the Genome Analysis Toolkit (GATK; https://www.broadinstitute.org/gatk/). The PacBio platform generated an average read length of 10 kb with 100 × coverage, while the DNBSEQ platform produced 150 bp paired-end reads with 50 × coverage. The final assembly consisted of a single circular chromosome with an N50 of 2.3 Mb and 99.5% completeness as assessed by BUSCO (Benchmarking Universal Single-Copy Orthologs, a tool for assessing genome assembly quality based on evolutionarily conserved orthologs).

### 2.5 Genome component prediction

Gene prediction for the *A. pleuropneumoniae strain* APPFJC001 genome was performed using Glimmer3 (http://www.cbcb.umd.edu/software/glimmer/) with Hidden Markov Models. Transfer RNA (tRNA), ribosomal RNA (rRNA), and small RNA (sRNA) genes were identified using tRNAscan-SE (Lowe and Eddy, 1997), RNAmmer, and the Rfam database, respectively. Tandem repeats were annotated using the Tandem Repeat Finder (http://tandem.bu.edu/trf/trf.html), with minisatellite and microsatellite DNA classified based on repeat unit length and number.

### 2.6 Gene annotation and protein classification

Functional annotation of predicted genes was performed using the BLAST alignment tool. Seven databases were utilized for general functional annotation: Kyoto Encyclopedia of Genes and Genomes (KEGG), Clusters of Orthologous Groups (COG), Non-Redundant Protein Database (NR), Swiss-Prot, Gene Ontology (GO), TrEMBL, and EggNOG. For pathogenicity and drug resistance analysis, virulence factors and antibiotic resistance genes were identified using the VFDB (Virulence Factors of Pathogenic Bacteria database) and ARDB (Antibiotic Resistance Genes Database), respectively.

### 2.7 Comparative genomics and hylogenetic analysis

To elucidate the phylogenetic relationships among *A. pleuropneumoniae* strains, a comprehensive genomic analysis was conducted using CorePan (a computational pipeline for core and pan-genome analysis that identifies conserved core genes across bacterial genomes and constructs phylogenetic trees based on concatenated core gene sequences). Core genes conserved across all selected strains were identified through comparative genomics [23]. Multiple sequence alignment of these core genes was performed using MAFFT with default parameters. For phylogenetic reconstruction, five reference strains (AP76, JL03, KL16, L20, and SAMN02469615; see Table 1 for strain details) were included for comparative genomic analysis. Two complementary approaches were applied: Topology construction: Maximum-likelihood trees (PHYML implementation in TreeBeST v1.9.2) were built under GTR + Γ model with 1000 bootstrap replicates [24]. Distance calculation: Pairwise SNP distances were derived from neighbor-joining algorithm (p-distance) applied to concatenated core-genome alignments. Core genes were defined as orthologous sequences present in all strains with ≥95% nucleotide identity over ≥80% gene length.

**Table 1:** Strains and genome sequences used in this study.

strain	serotype	Genome length(bp)	Coding sequences (total)	Accession no
JL03	Serotype 3	2,242,062	2036	CP000687
K16	Serotype 1	2,357,806	2168	CP022715
SAMN02469615	Serotype 1	2,318,649	2134	CP029003
L20	Serotype 5b	2,274,482	2012	CP000569

### 2.8 Pathogenicity test

Five 3-week-old susceptible commercial piglets were challenged intranasally with 2.5 × 10^9^ colony-forming units (CFU) of *A. pleuropneumoniae*. A control group was included for comparison. Following challenge, piglets were monitored daily for clinical symptoms over a 14-day period, as previously described [25]. Rectal temperatures were measured twice daily, with temperatures exceeding 41.8°C classified as high fever [26]. Piglets that showed severe respiratory distress were humanely euthanized for ethical considerations. Subsequently, lesions in the lungs, trachea, liver, spleen, and kidneys were examined. Tissue samples, comprising the heart, lung, trachea, liver, spleen, and kidneys, were collected for bacterial isolation and fixed in 3% formaldehyde solution for further analysis.

Methods of sacrifice: Piglets showing severe respiratory distress were humanely euthanized using CO_2_ asphyxiation followed by cervical dislocation to ensure complete cessation of vital signs. At the conclusion of the 14-day observation period, all surviving piglets were euthanized using the same protocol.

Methods of anesthesia and/or analgesia: No invasive procedures requiring anesthesia were performed during the study. The intranasal challenge was performed without anesthesia as it represents a minimally invasive procedure similar to natural infection routes.

Efforts to alleviate suffering: Animals were monitored twice daily for clinical signs. Environmental enrichment was provided, and piglets had ad libitum access to food and water. Body temperature monitoring was performed quickly to minimize stress. Any piglet showing severe distress was immediately euthanized to prevent prolonged suffering.

### 2.9 Histopathological examination

For comprehensive histopathological evaluation, tissue samples from multiple organs (including heart, liver, spleen, lung, kidney, and trachea) were aseptically collected from both infected and control groups. The collected specimens were immediately fixed in 10% neutral buffered formalin to ensure optimal tissue preservation. Following fixation, tissues were processed through a standardized dehydration series using graded ethanol solutions (70%, 80%, 90%, and 100%), cleared in xylene, and embedded in paraffin wax. Serial sections of 4–5 µm thickness were prepared using a rotary microtome and stained with hematoxylin and eosin (H&E) for routine histological examination. Histopathological assessment was conducted using light microscopy, with particular attention to lesion characterization and tissue morphology.

## 3 Results

### 3.1 Serotyping

Serotype identification of the *A. pleuropneumoniae* APPFJLYC01 strain was conducted by Zhaofeng Hua (Beijing) Biotechnology Co., Ltd. The results confirmed that the APPFJLYC01 strain belongs to serotype 1.

### 3.2 Characterization of the genome of strain APPFJLYC01

**Fig 1:**
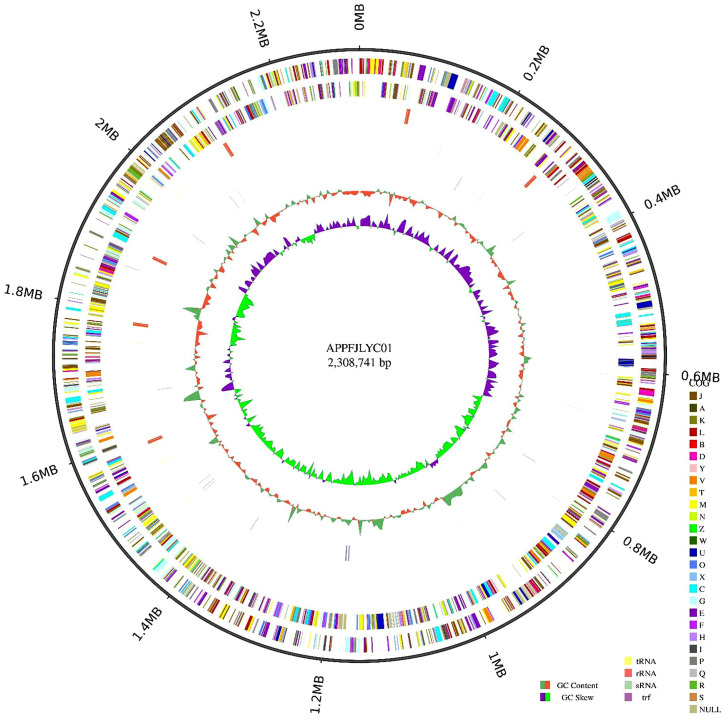
Gene circle: Genome circle of APPFJLYC01 strain. Note: the whole genome sequence of APPFJLYC01 strain was a circular chromosome with a total length of 2.3 mB. From the outside to the inside, they were the location coordinates of the genome sequence, the encoded genes, the results of gene function annotation, ncRNA, the GC content of the genome and the GC-skew value of the genome.

The complete genome of the APPFJLYC01 strain was sequenced and assembled, yielding a single circular chromosome with a total length of 2,308,741 base pairs (bp). The genome exhibited a GC content of 42.08% and contained 2,149 coding sequences (CDs), accounting for 87.60% of the total genome length. The total length of coding genes was 2,022,384 bp, with an average gene length of 941 bp. Tandem repeat analysis identified 65 tandem repeats (TRF), including 39 minisatellite DNAs and 4 microsatellite DNAs. Additionally, the genome harbored 64 tRNA genes, 19 rRNA genes (comprising 6 copies of 23S rRNA, 6 copies of 16S rRNA, and 7 copies of 5S rRNA), and 5 sRNA genes (Fig 1).

### 3.3 Genome functional analysis

#### 3.3.1 Functional annotation overview.

A total of 1,680, 1,530, 1,606, 1,880, and 2,143 genes were annotated in the KEGG, GO, Swiss-Prot, COG, and NR databases, respectively. The minimum number of annotated genes for any single function was 3.

#### 3.3.2 Gene ontology (GO) analysis.

**Fig 2:**
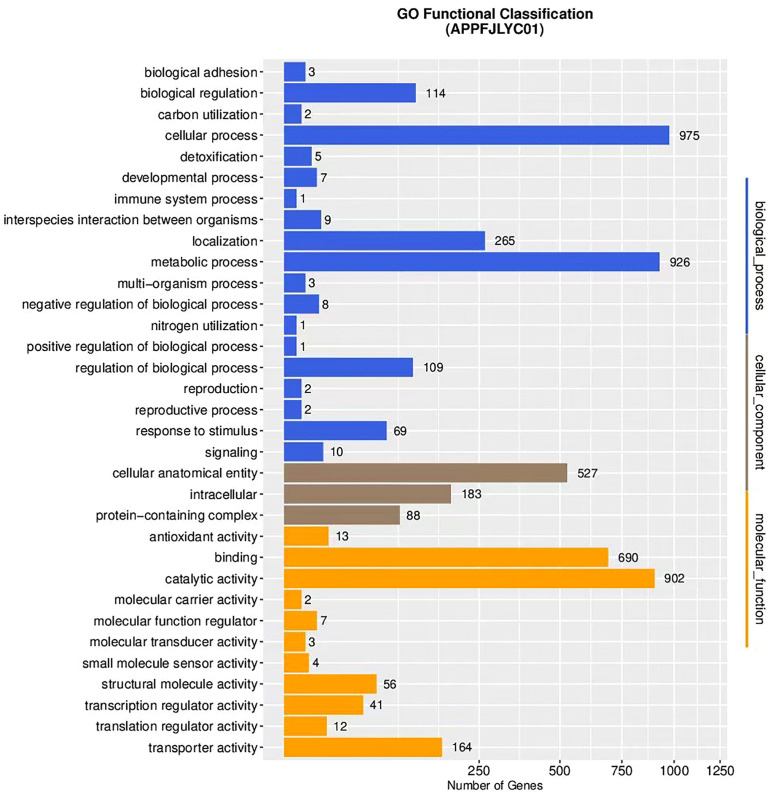
GO (Gene ontology) functional classification of strain APPFJLYC01: The figure illustrates the GO functional classification of genes from strain APPFJLYC01, categorized into Biological Process, Cellular Component, and Molecular Function. The x-axis represents the number of genes, and the y-axis lists the GO terms. Key categories include Metabolic process (926 genes), Cellular process (975 genes), and Localization (265 genes) under Biological Process; Intracellular (690 genes) and Protein-containing complex (902 genes) under Cellular Component; and Catalytic activity (164 genes) and Binding (250 genes) under Molecular Function.

A total of 1,530 genes were annotated and classified into three major ontologies and 33 functional sub-categories within the GO database. The proportion of genes annotated to biological processes, cellular components, and molecular functions was 48.3%, 15.3%, and 36.4% respectively. With regard to the biological process ontology, the majority of genes are involved in cellular and metabolic processes. In the molecular function ontology, genes related to binding and catalytic activities are predominant. For the cellular component ontology, genes associated with cellular structural entities are the most prevalent. The detailed distribution of GO annotations and the GO-based functional classification of genes from strain APPFJLYC01 are presented in Fig 2. In this figure, the x-axis represents the gene count, while the y-axis lists the GO terms. Under the biological process ontology, key sub-categories include the metabolic process (926 genes), cellular process (975 genes), and localization (265 genes). For the cellular component ontology, the major sub-categories are intracellular (690 genes) and protein-containing complex (902 genes). In the molecular function ontology, the main sub-categories are catalytic activity (164 genes) and binding (250 genes).

#### 3.3.3 KEGG pathway analysis.

**Fig 3:**
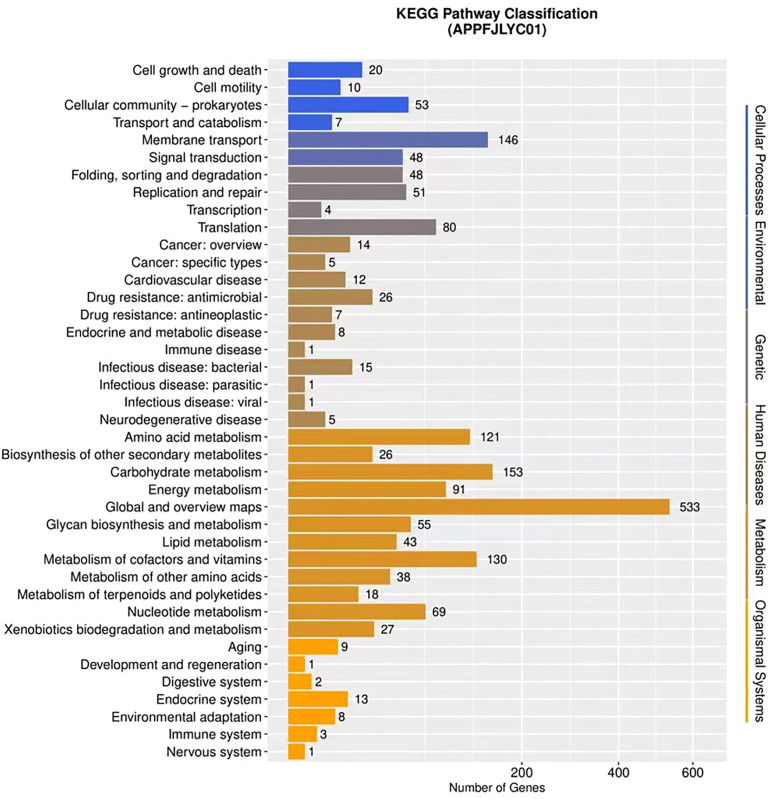
KEGG pathway classification of strain APPFJLYC01: The figure presents the KEGG pathway classification of genes from strain APPFJLYC01. The x-axis represents the number of genes, and the y-axis lists the KEGG pathway categories. Key pathways include Carbohydrate metabolism (153 genes), Energy metabolism (91 genes), Amino acid metabolism (121 genes), and Membrane transport (146 genes). Other notable pathways are Signal transduction (48 genes), Replication and repair (51 genes), and Translation (80 genes).

A total of 1,680 orthologous protein-coding genes were mapped to 6 major categories of KEGG metabolic pathways. The pathways with the highest gene representation were metabolism (68.8%) and environmental adaptation (10.2%), as shown in Fig 3. These pathways are essential for sustaining bacterial metabolic activities and survival.

#### 3.3.4 Clusters of orthologous groups (COG) analysis.

**Fig 4:**
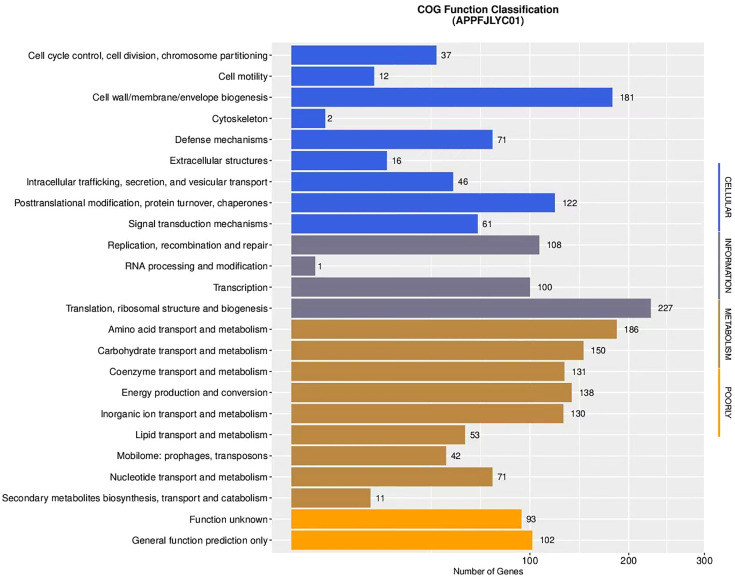
COG functional classification analysis of strain APPFJLYC01: The figure illustrates the functional classification of genes from strain APPFJLYC01 based on the COG database. The x-axis represents the number of genes, and the y-axis lists the COG functional categories. Key categories include Translation, ribosomal structure and biogenesis (227 genes), Amino acid transport and metabolism (186 genes), and Carbohydrate transport and metabolism (150 genes). Other notable categories are Energy production and conversion (138 genes), Replication, recombination and repair (108 genes), and Signal transduction mechanisms (61 genes).

The COG analysis annotated 1,880 genes, which were classified into 20 functional categories (C–V). The predominant functional categories included Ribosome biosynthesis, Biofilm formation, and Amino acid transport and metabolism. The results of the COG analysis were consistent with the KEGG pathway findings, highlighting the involvement of numerous genes in metabolic processes essential for bacterial survival. A detailed breakdown of COG functional categories is presented in Fig 4.

#### 3.3.5 Virulence factors and antibiotic resistance genes.

i) Virulence actors Gene Analysis

A total of 190 non-redundant virulence genes were identified and functionally annotated in the APPFJLYC01 strain using DIAMOND analysis against the Virulence Factor Database (VFDB). Positive matches were defined by a sequence identity of ≥40% and an e-value threshold of <1e-^10^, followed by manual curation to remove duplicate entries and validate annotations. The most abundant virulence factors were associated with immune modulation (67 genes, 35.3%) and nutritional/metabolic adaptation (49 genes, 25.8%) (Table 2).

**Table 2:** Distribution of putative virulence genes identified in APPFJLYC01 strain using VFDB analysis.

Category of Virulence Factor	Number of Genes	Percentage (%)
Immune modulation	67	35.3
Nutritional/Metabolic factor	49	25.8
Adherence	35	18.4
Exotoxin	9	4.7
Regulation	7	3.7
Stress survival	7	3.7
Effector delivery system	6	3.2
Biofilm	5	2.6
Motility	2	1.1
Post-translational modification	2	1.1
Exoenzyme	1	0.5
**Total**	**190**	**100**

Gene counts reflect non-redundant annotations after manual validation. Full gene annotations are available in Supplementary S1 Table.[Subxref1]

Further analysis revealed that the virulence repertoire encompassed genes mediating adherence (35 genes), exotoxin production (9 genes), and regulatory functions. Notably, 4 adhesion-related virulence factors (e.g., *comE, flpD*) exhibited sequence identities exceeding 99% to reference pathogens. Key exotoxin genes included *hlyA, hlyB, hlyC, hlyD*, and *argK*. A complete list of annotated virulence genes per category is provided in Supplementary S1 Table.[Subxref1]

ii) Antibiotic Resistance Gene Analysis

Comparative analysis of the bacterial genome against the Antibiotic Resistance Genes Database (ARDB) identified 10 putative antibiotic resistance genes with significant matches (E-value < 1e-5, identity > 40%). These genes confer resistance to multiple antibiotic classes, including β-lactams, tetracyclines, aminoglycosides, macrolides, and others, through mechanisms such as drug efflux, enzymatic modification, and target modification (Table 3). These findings highlight the multidrug resistance potential of the APPFJLYC01 strain, with resistance mechanisms spanning multiple antibiotic classes.

**Table 3:** Summary of identified antibiotic resistance genes.

Gene ID	Resistance Type	Antibiotic Class	Mechanism	Identity (%)	E-value
APPFJLYC01GL000203	*pbp1a*	Penicillin	PBP modification	62.1	1.60E-301
APPFJLYC01GL000268	*tet34*	Tetracycline	Ribosomal protection protein – confers resistance by protecting ribosomes from tetracycline binding	61.3	3.30E-56
APPFJLYC01GL000387	*norm*	Tigecycline, Streptomycin, Kanamycin, Ciprofloxacin, Norfloxacin	Efflux pump (MATE family)	46.7	5.80E-109
APPFJLYC01GL000418	*ksga*	Kasugamycin	Ribosomal methylation	67.6	2.00E-104
APPFJLYC01GL000578	*mexw*	Multidrug	Efflux pump (RND system)	44.9	7.50E-258
APPFJLYC01GL000939	*dfra26*	Trimethoprim	Drug-insensitive DHFR	43.0	1.80E-33
APPFJLYC01GL001677	*pbp2*	Penicillin	PBP modification	48.7	1.10E-182
APPFJLYC01GL001685	*emre*	Aminoglycosides	Efflux pump	43.8	1.80E-23
APPFJLYC01GL001909	*macb*	Macrolides	Efflux pump (RND system)	48.3	4.20E-174
APPFJLYC01GL001919	*pbp1b*	Penicillin	PBP modification	42.4	4.10E-171

#### 3.3.6 Analysis of core and strain-specific genes.

**Fig 5:**
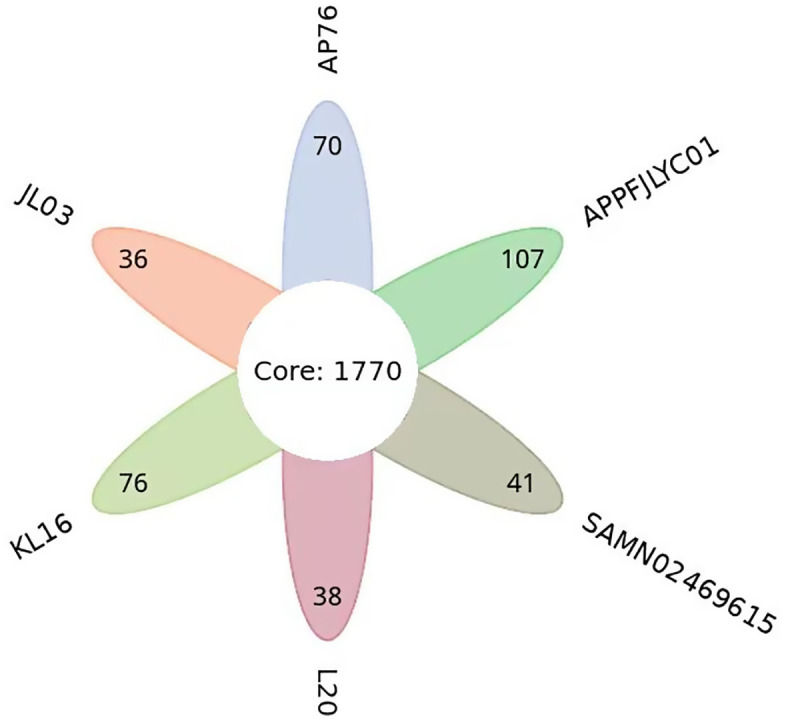
Comparative pan-genome analysis of APPFJLYC01 and reference strains. The Venn diagram illustrates the comparative pan-genome analysis of the APPFJLYC01 strain and five reference strains (AP76, JL03, KL16, L20, and SAMN02469615). The analysis identified 1,770 core genes shared among all strains, representing conserved genomic regions. The APPFJLYC01 strain exhibited 107 strain-specific genes, the highest number among the compared strains, indicating unique genetic features that may contribute to its distinct phenotypic characteristics.

A comparative analysis of the pan-genome was conducted among the APPFJLYC01 strain and five reference strains (AP76, JL03, KL16, L20, and SAMN02469615). The Venn diagram (Fig 5) revealed that these strains share 1,770 core genes, representing conserved genomic regions. Notably, the APPFJLYC01 strain exhibited 107 strain-specific genes, the highest number among the compared strains, suggesting unique genetic features that may contribute to its distinct phenotypic characteristics.

#### 3.3.7 Gene family analysis.

**Fig 6:**
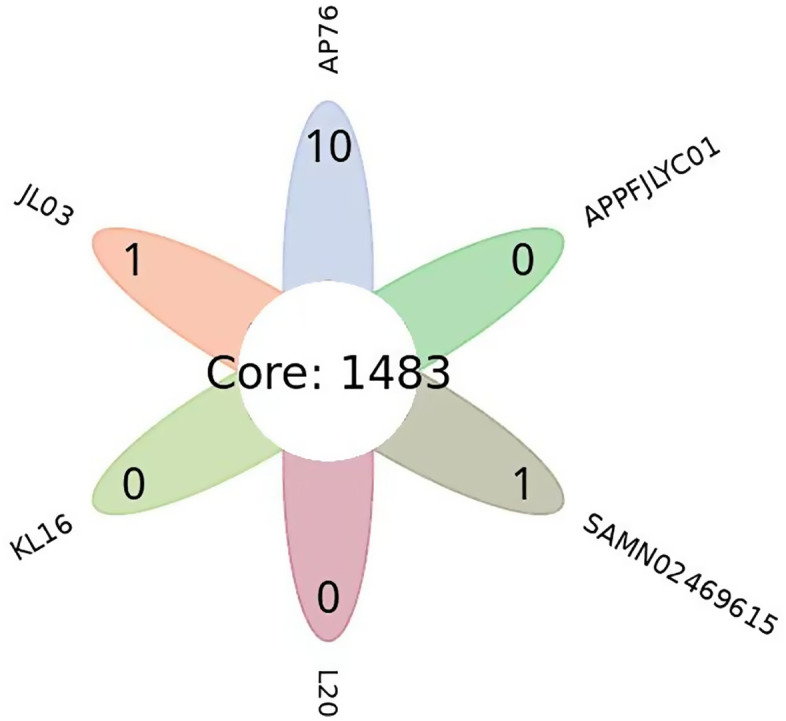
Venn diagram of homologous gene families in APPFJLYC01 and reference strains: The venn diagram displays the distribution of homologous gene families among the APPFJLYC01 strain and reference strains (AP76, JL03, KL16, L20, and SAMN02469615). Each ellipse represents a strain or group of strains. The numbers in each region indicate the count of gene families unique to that region, while the central overlapping region represents 1,483 core gene families shared by all strains.

Gene family analysis was performed to compare the APPFJLYC01 strain with the five reference strains (JL03, KL16, L20, AP76, and SAMN02469615). The results showed that The APPFJLYC01 strain contains 2,149 genes, while the KL16 strain has the highest gene count (2,209 genes). The APPFJLYC01 strain comprises 1,635 gene families, with 0 strain-specific gene families identified (Table 4). A total of 1,483 gene families were shared among all six strains, indicating a high degree of genomic conservation (Fig 6). These findings highlight the genetic diversity and evolutionary relationships among the analyzed strains, with APPFJLYC01 exhibiting a unique gene repertoire despite sharing a substantial number of conserved gene families with other strains.

**Table 4:** Statistical table of gene families.

Strain number	Number of genes	Number of genes (aggregating)	Number of genes (not aggregating)	Number of gene families	Number of endemic gene families
AP76	2142	2092	50	1658	10
JL03	2036	2005	31	1622	1
KL16	2209	2135	74	1710	0
L20	2012	1986	26	1596	0
SAMN02469615	2077	2039	38	1673	1
APPFJLYC01	2149	2038	111	1635	0

### 3.4 Phylogenetic analysis

**Fig 7:**
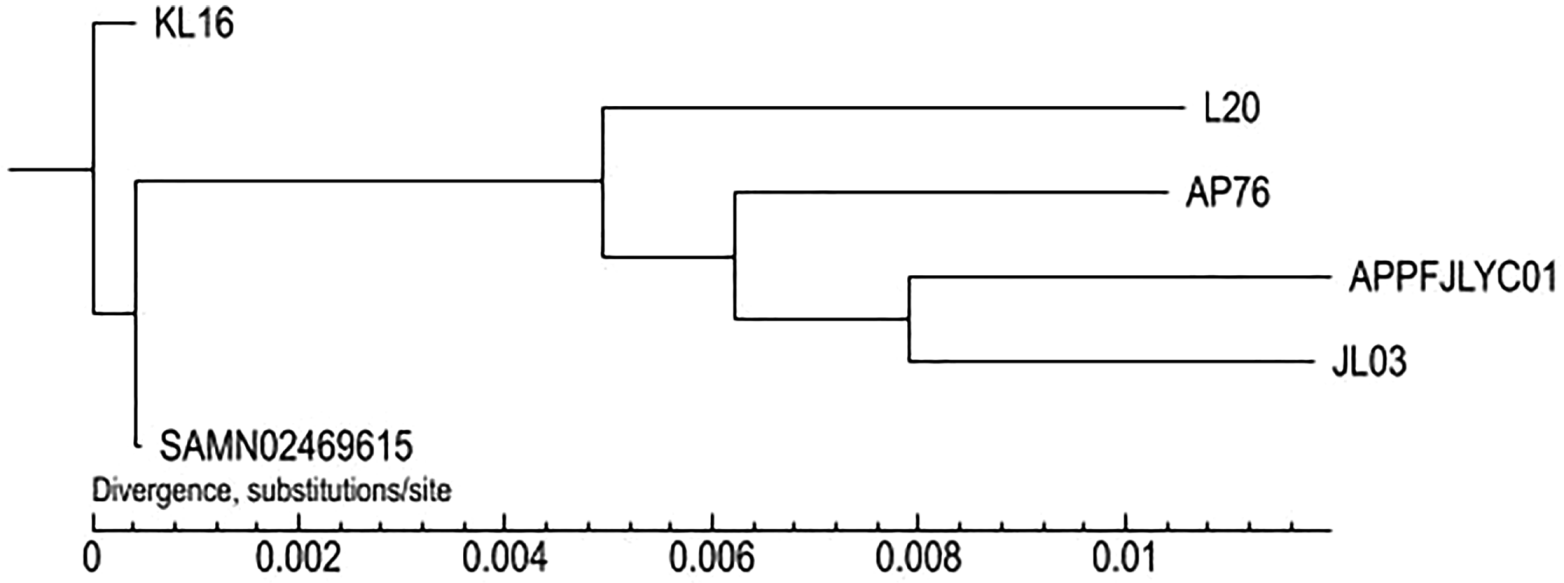
Maximum-likelihood phylogenetic tree (constructed with TreeBeST v1.9.2) depicting evolutionary relationships among APPFJLYC01 and five reference strains, where APPFJLYC01 and serotype 1 strain JL03 form a strongly supported monophyletic clade (98% bootstrap, red highlight). While serotype 1 strain AP76 occupies an earlier branching position than JL03, pairwise SNP analysis identifies it as the second closest relative to APPFJLYC01 after JL03 based on genetic distances (scale bar: substitutions/site). Divergence levels show L20 (serotype 5b) with moderate separation, and serotype 1 strains KL16/SAMN02469615 exhibiting greater distances.

Phylogenetic reconstruction based on CorePan analysis revealed distinct evolutionary relationships among APPFJLYC01 and five reference strains (AP76, JL03, KL16, L20, SAMN02469615). APPFJLYC01 and serotype 1 strain JL03 formed a well-supported monophyletic clade (98% bootstrap support), consistent with their shared geographical origin as Chinese porcine isolates. Despite occupying an earlier branching position in the phylogenetic tree, serotype 1 strain AP76 demonstrated the second closest genetic relationship to APPFJLYC01 based on pairwise SNP distances. Divergence analysis further showed that while L20 (serotype 5b) exhibited moderate separation, serotype 1 strains KL16 and SAMN02469615 displayed greater genetic distances, suggesting complex evolutionary dynamics within this serogroup. The complete phylogenetic relationships are presented in Fig 7.

### 3.5 Clinical symptoms and pathologicalc changes in piglets

#### 3.5.1 Clinical symptoms.

Following experimental challenge, piglets in the treatment group exhibited pronounced clinical manifestations indicative of systemic illness. The most prominent clinical sign was pyrexia, characterized by a mean temperature elevation of 2.5°C (range: 39.5–41.5°C) compared to the normal physiological range of 38–39.5°C. Behavioral changes included marked lethargy, reduced mobility, and characteristic huddling behavior, suggesting thermoregulatory distress and systemic discomfort. Nutritional intake was significantly impaired, physical examination revealed poor body condition scores, characterized by rough hair coats and decreased skin elasticity. Respiratory distress was evident in all affected animals, manifested as dyspnea and increased respiratory rates.

Gastrointestinal disturbances were observed in 40% of challenged piglets, presenting as acute vomiting and watery diarrhea. These clinical manifestations contributed to rapid body weight loss, with affected piglets losing 20% of their initial body weight within 72 hours post-challenge. The constellation of clinical signs indicated severe systemic involvement and rapid disease progression in the experimental group.

#### 3.5.2 Pathological changes.

**Fig 8:**
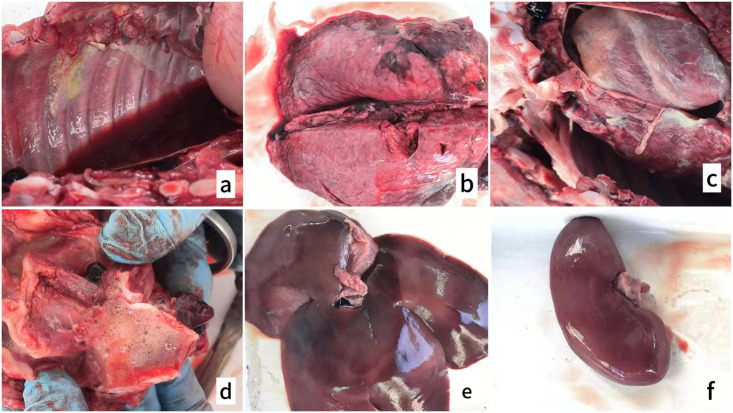
Pathological lesions in piglets succumbing to Actinobacillus pleuropneumoniae infection. Various pathological changes observed in pigs infected with APPFJLYC01. **(a)** Pleural and pulmonary adhesions in the thoracic cavity, accompanied by yellow fibrinous exudate, indicating a strong inflammatory response. **(b)** Severe congestion and edema in the lungs, with multifocal nodules of varying sizes, suggesting localized areas of intense inflammation and tissue damage. **(c)** Enlarged heart with fibrinous exudate on the surface, consistent with characteristic “villous heart” lesions commonly associated with A. pleuropneumoniae infection. **(d)** Congested and swollen trachea, with abundant mucus accumulation in the lumen, impairing normal respiratory function. **(e)** Greenish discoloration and severe peripheral congestion in the liver, indicating compromised metabolic and detoxification functions. **(f)** Congested spleen and swollen kidneys, suggesting systemic inflammation and potential renal damage.

Necropsy of piglets that succumbed to the challenge revealed a spectrum of pathological features consistent with severe *A. pleuropneumoniae* infection. In the thoracic cavity, pleural and pulmonary adhesions were observed, accompanied by yellow fibrinous exudate, indicating a robust inflammatory response (Fig 8a). The lungs displayed severe congestion and edema, with multifocal nodules of varying sizes on the surface, suggestive of localized areas of intense inflammation and tissue damage (Fig 8b). Additionally, the hilar lymph nodes were congested and swollen, reflecting the involvement of the lymphatic system in the immune response. The heart appeared notably enlarged, with fibrinous exudate on its surface, which is characteristic of “villous heart” lesions commonly associated with *A. pleuropneumoniae* infection (Fig 8c). The trachea exhibited congestion and swelling, with abundant mucus accumulation in the lumen, which impaired normal respiratory function (Fig 8d). In the abdominal cavity, the liver showed a greenish discoloration and severe peripheral congestion, suggesting compromised metabolic and detoxification functions (Fig 8e). The spleen was congested, likely due to an increased immune response, while the kidneys were swollen, indicating systemic inflammation and potential renal damage (Fig 8f). Collectively, these findings underscore the profound systemic impact of APPFJLYC01 infection, highlighting its severe effects on both thoracic and abdominal organs.

#### 3.5.3 Histological findings associated with APPFJLYC01 infection.

**Fig 9:**
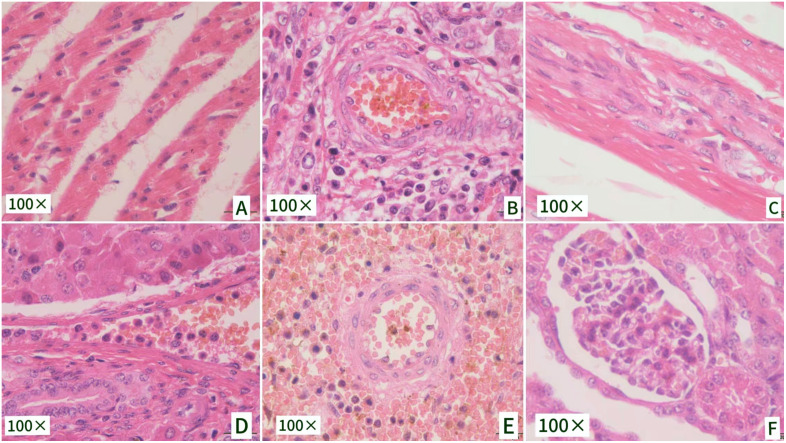
Histopathological examination of tissue sections from piglets infected with Actinobacillus pleuropneumoniae. The figure presents histopathological observations of tissue sections from piglets infected with Actinobacillus pleuropneumoniae, revealing cellular-level damage caused by the infection.

Histological analysis of tissue sections from infected piglets revealed pathological features consistent with *A. pleuropneumoniae* infection. In the thoracic tissue, severe disruption of the normal tissue architecture was observed, with prominent edema and inflammatory cell infiltration (Fig 9A). This suggests the acute inflammatory response typically triggered by *A. pleuropneumoniae*, which induces significant tissue damage and immune activation. The presence of central necrosis surrounded by inflammatory cells was evident in multiple organs (Fig 9B), with neutrophils and macrophages concentrated around areas of tissue destruction. These findings are indicative of the acute stage of infection, where *A. pleuropneumoniae* causes widespread tissue necrosis and an intense local immune response [13]. Additionally, fibrosis and tissue remodeling were observed in trachea (Fig 9C)., suggesting progression to the chronic phase of the infection. The persistent inflammatory response likely leads to ongoing tissue damage and repair attempts, a hallmark of chronic *A. pleuropneumoniae* infections [27]. Further, the formation of abscesses, surrounded by inflammatory infiltrates, was apparent in spleen (Fig 9E), consistent with abscess formation observed in *A. pleuropneumoniae* lesions. The necrotic tissue and surrounding inflammation in liver (Fig 9D) and kiney(Fig 9F) further corroborate the severe lung damage and systemic effects typically associated with *A. pleuropneumoniae* infections, including the formation of localized abscesses. These histological findings collectively highlight the profound tissue damage caused by *A. pleuropneumoniae* infection, characterized by acute necrosis, abscess formation, and the progression to chronic inflammation and fibrosis.

## 4 Discussion

This study on the *A. pleuropneumoniae* strain APPFJLYC01 offers a comprehensive understanding of its genomic features, virulence factors, antibiotic resistance profile, and pathogenicity, with significant implications for porcine pleuropneumonia research. The genome of APPFJLYC01, comprising 2,308,741 bp with a GC content of 42.08%, encodes 2,149 genes, including tRNAs, rRNAs, and sRNAs. These characteristics align with those of other *A. pleuropneumoniae* strains [27]. Functional annotation using KEGG, GO, and COG databases revealed strong metabolic capabilities, particularly in transcription, translation, and biofilm formation. Biofilm formation is a key survival mechanism for bacteria in the host environment [28].

Virulence factor analysis identified 190 putative virulence genes, including those involved in adhesion, immune modulation, and exotoxin production [10–12,29]. Notably, four adhesion-related genes exhibited >99% homology, suggesting their crucial role in host colonization [12]. The high conservation of these adhesion factors indicates strong selective pressure for maintaining host-pathogen interactions, which is critical for bacterial survival and persistence in the swine respiratory tract. These adhesins likely include type IV pili components, outer membrane proteins, and surface lipoproteins that facilitate initial bacterial attachment to epithelial cells and subsequent colonization of the respiratory mucosa.

The diversity of adhesion mechanisms identified in APPFJLYC01 suggests a multi-layered approach to host colonization. The *comE* gene, involved in competence development, may facilitate horizontal gene transfer and adaptation to host environments. The *gapA* gene encoding glyceraldehyde-3-phosphate dehydrogenase serves dual functions as both a metabolic enzyme and a surface-exposed adhesin that binds to host cell receptors. The *tad* (tight adherence) gene cluster (*tadA-tadG*) is particularly significant as it encodes the machinery for type IVb pili assembly, which is essential for biofilm formation and persistent colonization. The flp (fimbrial low-molecular-weight protein) genes (*flpB, flpC, flpD*) work in conjunction with the tad genes to form the pilus structure, creating a robust attachment system that resists host clearance mechanisms.

Additionally, hemolytic exotoxin genes (*hlyA, hlyB, hlyC, hlyD, and argK*) showed homology ranging from 42.4% to 86.1%, indicating potential genetic recombination events. The variable homology of these exotoxin genes suggests ongoing evolutionary pressure to diversify toxin repertoires, potentially to evade host immune responses or to adapt to different host environments. The hlyCABD operon encodes the RTX (Repeats-in-Toxin) hemolytic toxin system, where *hlyA* codes for the toxin structural gene, *hlyB* and *hlyD* encode transport proteins, and *hlyC* encodes the acylation enzyme necessary for toxin activation. The *argK* gene, encoding ornithine carbamoyltransferase, is crucial for arginine metabolism and has been linked to enhanced virulence through metabolic adaptation in nutrient-limited host environments.

The strain also harbors numerous immune-modulatory virulence factors, which likely influence host immune responses and contribute to bacterial [19]. The iron acquisition systems identified in APPFJLYC01, including the *hemE, hemA, hemC, hemD, hemG*, and *hemH* genes involved in heme biosynthesis, and the *fetA* gene encoding an iron transporter, are crucial for bacterial survival in the iron-limited host environment. Iron is essential for bacterial metabolic processes, and the ability to compete effectively with host iron-binding proteins (transferrin, lactoferrin) determines bacterial fitness. The *exbB-exbD* genes encode the TonB energy transduction system, which provides energy for active transport of iron-siderophore complexes across the outer membrane. The *hitC* gene likely encodes a component of the iron transport system specific for heme uptake, allowing the bacterium to utilize host hemoglobin as an iron source during infection.

The antibiotic resistance profile of *A. pleuropneumoniae* is critical for guiding antibiotic use in the prevention and treatment of porcine pleuropneumonia. Ten resistance genes were identified, with homology ranging from 42.2% to 67.6%. The *ksgA* gene, conferring resistance to kasugamycin, exhibited the highest homology [18]. The *ksgA* gene encodes 16S rRNA dimethyltransferase, which modifies specific adenine residues in the 16S rRNA, thereby preventing kasugamycin binding to the ribosome. This modification not only confers resistance to kasugamycin but may also affect ribosomal accuracy and translation efficiency, potentially influencing bacterial fitness and virulence expression.

Whole genome sequencing (WGS) has proven effective in identifying antimicrobial resistance (AMR) genes in *A. pleuropneumoniae*, with studies linking elevated minimum inhibitory concentrations (MICs) to the presence of specific AMR genes. For instance, tetracycline resistance genes such as *tet34* gene are commonly found in *A. pleuropneumoniae* isolates and are associated with resistance to tetracycline. This enzymatic modification of tetracycline molecules renders them inactive, providing a biochemically distinct resistance pathway that may be less susceptible to efflux pump inhibitors.

Moreover, the multidrug efflux pumps identified in APPFJLYC01, including the *norM* gene (encoding a MATE family transporter) and *mexW* (encoding an RND system component), represent particularly concerning resistance mechanisms due to their broad substrate specificity. The *norM* gene confers resistance to multiple antimicrobial classes including fluoroquinolones (ciprofloxacin, norfloxacin), aminoglycosides (streptomycin, kanamycin), and tigecycline through active efflux. This multidrug resistance transporter can potentially export newly developed antimicrobials, limiting treatment options. The *mexW* gene, part of the RND (Resistance-Nodulation-Division) efflux system, works synergistically with membrane fusion proteins and outer membrane channels to create a tripartite efflux system capable of expelling a wide range of antimicrobials across the bacterial cell envelope.

The overuse or misuse of antibiotics can select for resistant strains, complicating disease control efforts. The presence of resistance genes with low homology suggests potential genetic recombination events that may have shaped the strain’s resistance profile, possibly leading to novel resistance mechanisms [13]. This resistance profile necessitates careful antimicrobial stewardship and highlights the urgent need for novel therapeutic approaches, including combination therapies with efflux pump inhibitors, alternative antimicrobial agents, or immunomodulatory treatments.

Comparative genomic analysis revealed 1,770 shared core genes essential for the survival and function of *A. pleuropneumoniae*. The 107 unique genes identified in APPFJLYC01, the highest number among the compared strains, suggest that this strain may possess unique characteristics. Phylogenetic analysis places APPFJLYC01 and strain JL03 on the same branch, indicating a close evolutionary relationship. This genetic similarity may reflect common selective pressures or geographic origin [30]. The identification of potential genetic variations in virulence genes related to metabolism, adhesion, and colonization suggests that these genes may have evolved differently among strains, contributing to variations in pathogenicity. As highlighted by Bosse et al. profile [13], genetic variations in virulence genes can alter host-pathogen interactions, leading to diverse disease outcomes. Genomic comparative analysis provides valuable insights into the transmission and epidemiological patterns of *A. pleuropneumoniae*, suggesting vertical transmission, including resistance genes, within integrated systems [31].

The pathological and histological findings from *A. pleuropneumoniae* infection in piglets highlight the severe impact of this pathogen on both local and systemic tissues. The clinical symptoms, including elevated body temperature, respiratory distress, and severe pleural and pulmonary adhesions, are consistent with previous reports on *A. pleuropneumoniae* infections [26,32]. The disease can present in acute or chronic forms, with acute infections characterized by dyspnea, high fever, and bloody nasal discharge, and chronic cases exhibiting less specific symptoms [33]. In experimental settings, *A. pleuropneumoniae* serotype 9 induces severe clinical signs and lung lesions, particularly in younger pigs [34]. Histologically, the infection leads to fibrinohemorrhagic pleuropneumonia, marked by extensive hemorrhage, necrosis, fibrin deposition, and abscesses in the lungs [35]. The present study observed similar lesions, including central necrosis surrounded by inflammatory infiltrates, abscess formation, and tissue disruption. These acute-stage features reflect intense tissue destruction and a robust immune response, leading to necrosis and abscess formation [13]. The fibrosis observed in later-stage lesions highlights the chronic nature of unresolved infections, with immune-mediated scar tissue formation [27]. This progression from acute to chronic inflammation, along with systemic involvement of organs such as the liver and kidneys, underscores the complexity and severity of *A. pleuropneumoniae* infections.

The identification of 190 virulence genes, including those related to adhesion, immune modulation, and exotoxin production, provides a molecular basis for the pathogenicity of APPFJLYC01. The high homology of four adhesion-related genes (>99%) suggests that adhesion is a critical step in host colonization, preventing clearance by the immune system Jacques [12]. The variable homology of hemolytic exotoxin genes (42.4% − 86.1%) may reflect genetic recombination events that alter the spectrum and toxicity of these exotoxins, potentially enhancing bacterial virulence [36].

The discovery of unique genes and genetic variations in APPFJLYC01 underscores the importance of continued surveillance and research. Future studies should focus on elucidating the functions of these unique genes, using gene knockout or overexpression techniques to investigate their roles in virulence, metabolism, and antibiotic resistance. These genes may serve as potential targets for novel therapeutics or vaccine development. Additionally, longitudinal studies in different regions could provide insights into the evolution of *A. pleuropneumoniae* strains, the spread of resistance genes, and the emergence of more virulent strains. This information is crucial for the development of targeted prevention and control strategies to mitigate the economic losses caused by porcine pleuropneumonia in the swine industry.

## Supporting information

S1 Table Curated list of 190 virulence genes in APPFJLYC01 strain. (DOCX)
