# Supplementary material for: Genome analysis of Actinobacillus pleuropneumoniae strain APPFJLYC01 reveals multidrug resistance and high virulence potential
Source: PLoS One. 2025 Nov 14;20(11):e0336060. doi: 10.1371/journal.pone.0336060 (PMC12617859; doi:10.1371/journal.pone.0336060)
Supplement: S1 Table — (DOCX) [file pone.0336060.s001.docx]

**Supplementary Material**

Genome analysis of *Actinobacillus pleuropneumoniae* strain APPFJLYC01 reveals multidrug resistance and high virulence potential

**Fang Zhihong^1^, Lin Zecheng^1^,** **Duan Chuchu^2^, Liu Xiaojin^1^, Luo Zhongfeng^1^,** **Huang Cuiqin^1,3,4^, Li Xiaohua^1,3,4,^ * , Zheng Xintian^1,3,4,^ ***

1 College of Life Science, Longyan University, Longyan 364012, China;

2 College of Animal Science, Fujian Agriculture and Forestry University, Fuzhou 350000, China;

3 Fujian Provincial Key Laboratory of Animal Infectious Disease Control and Biotechnology, Longyan 364012, China;

4 Engineering Research Center of Zoonotic Diseases Prevention and Control, Fujian Universities, Longyan 364012, China;

* Correspondence and reprint requests: Zheng Xintian, Li Xiaohua. E-mail: [xintianzheng@lyun.edu.cn,](mailto:xintianzheng@lyun.edu.cn,) xh_li276@163.com

## Keywords: *Actinobacillus pleuropneumoniae suis*, Whole gene sequence analysis, Bioinformatics analysis, pathogenicity

**Table S1. Curated list of 190 virulence genes in APPFJLYC01 strain**

| Category | Number of Genes | Gene Names |
| --- | --- | --- |
| Adherence | 35 | *comE, gapA, tadA, tadB, tadC, tadD, tadE, tadF, tadG, flpD, rcpB, rcpA, flpC, flpB, flp-1, IlpA, dnaK, htpB, pomA, oapA, tufA, lap, pilQ, HMW1, HMW2, vfr, ML_RS08565, DNO_RS02105, APL_RS04580, APL_RS04585, APL_RS04590, APL_RS04595, dnaK, htpB, pomA, tufA* |
| Biofilm | 5 | *algU, algW, luxS, mucD, pgaC* |
| Effector delivery system | 6 | *LPG_RS14840, rimP, hrpB, clpV, CBU_1566, CT_473* |
| Exoenzyme | 1 | *eno* |
| Exotoxin | 9 | *hlyA (GL000236), hlyA (GL001012), argK, hlyD (GL001391), hlyB (GL001392), hlyA (GL001393), hlyC, hlyD (GL001463), hlyB (GL001464)* |
| Immune modulation | 67 | *LpxB, lpxC, oppF, kdsB, licA, rfaC, kpsF, lpxA, fabZ, lpxD, lgtF, waaQ, cpsA, orfM, manB, ompP2, msbA, gmhA, gmhB, htrB, kdkA, lsgD, lsgE, lsgF, kdtA, kdtB, adhD, lpxK, galE, gndA, rmlB, weeC, wbjD, weeA, bexD, bexC, bexB, bexA, rfaD, lpxH, acpXL, rpe, oppF, gmhA, msbA, kpsF, fabZ, cpsA, manB, msbA, galE, gndA, adhD, lpxK, kdtA, kdtB, rfaD, lpxH, weeC, wbjD, weeA, bexD, bexC, bexB, bexA, hcsA, hcsB, glf, pgi, manA, glmM* |
| Motility | 2 | *tcyJ, flmH* |
| Nutritional/Metabolic factor | 49 | *hemE, bioB, sitB, sitA, sugC, qbsC, hemA, vctD, bauE, hemM, hemY, hemX, hemD, hemC, ccmF, hutZ, putM, fetA, hitC, ccmE, ccmC, ccmB, hemL, exbD, exbB, hemG, irp6C, hemH, putC, hemN, leuD, purM, chuW, TbpAB, exbD, exbB, hemB, purC, glnA1, diaminobutyrate, Hgp, mbtI, Achromobactin, sitB, sitA, sugC, hemA, hemM, hemY, hemX, hemD, hemC* |
| Post-translational modification | 2 | *lspA, mip* |
| Regulation | 7 | *relA, regX3, mprA, sigA, fur, relA, sigA* |
| Stress survival | 7 | *sodCI, sodB, katA, ureG, ureB, ureA, clpP* |
